# Toughening of a Soft Polar Polythiophene through Copolymerization with Hard Urethane Segments

**DOI:** 10.1002/advs.202002778

**Published:** 2020-12-11

**Authors:** Sepideh Zokaei, Renee Kroon, Johannes Gladisch, Bryan D. Paulsen, Wonil Sohn, Anna I. Hofmann, Gustav Persson, Arne Stamm, Per‐Olof Syrén, Eva Olsson, Jonathan Rivnay, Eleni Stavrinidou, Anja Lund, Christian Müller

**Affiliations:** ^1^ Department of Chemistry and Chemical Engineering Chalmers University of Technology Göteborg 41296 Sweden; ^2^ Laboratory of Organic Electronics Department of Science and Technology Linköping University Norrköping 60174 Sweden; ^3^ Wallenberg Wood Science Center Department of Science and Technology Linköping University Norrköping 60174 Sweden; ^4^ Department of Biomedical Engineering Northwestern University Evanston IL 60208 USA; ^5^ Department of Physics Chalmers University of Technology Göteborg 41296 Sweden; ^6^ Department of Fibre and Polymer Technology KTH Royal Institute of Technology Stockholm 11428 Sweden; ^7^ Wallenberg Wood Science Center KTH Royal Institute of Technology Stockholm 11428 Sweden; ^8^ Wallenberg Wood Science Center Chalmers University of Technology Göteborg 41296 Sweden

**Keywords:** chemical and electrochemical doping, organic electrochemical transistors (OECT), polar conjugated polymers, swelling, urethane

## Abstract

Polar polythiophenes with oligoethylene glycol side chains are exceedingly soft materials. A low glass transition temperature and low degree of crystallinity prevents their use as a bulk material. The synthesis of a copolymer comprising 1) soft polythiophene blocks with tetraethylene glycol side chains, and 2) hard urethane segments is reported. The molecular design is contrary to that of other semiconductor‐insulator copolymers, which typically combine a soft nonconjugated spacer with hard conjugated segments. Copolymerization of polar polythiophenes and urethane segments results in a ductile material that can be used as a free‐standing solid. The copolymer displays a storage modulus of 25 MPa at room temperature, elongation at break of 95%, and a reduced degree of swelling due to hydrogen bonding. Both chemical doping and electrochemical oxidation reveal that the introduction of urethane segments does not unduly reduce the hole charge‐carrier mobility and ability to take up charge. Further, stable operation is observed when the copolymer is used as the active layer of organic electrochemical transistors.

## Graphical abstract

A soft polythiophene with tetraethylene glycol side chains is copolymerized with hard urethane blocks. The copolymer exhibits improved ductility and stiffness, owing to a reversible network formed through hydrogen‐bonding between urethane segments, while electrical and electrochemical properties are retained to a great extent. The hydrogen‐bonded network reduces the degree of swelling of the copolymer as compared to the homopolymer.

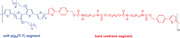

## 1 Introduction

Conjugated polymers that are able to deform without fracture are highly sought after for the realization of truly flexible electronic devices.^[^ ^1^, ^2^, ^3^, ^4^, ^5^ ^]^ A polymer will only be able to accommodate a high degree of strain if its glass transition temperature *T*_g_ lies well below the operating temperature of the target application. At the same time, the material should behave like a solid and not a viscous substance, which requires reinforcement, for instance through crystallites or other types of physical or chemical crosslinks.

The rigid backbone of conjugated polymers, which often comprises planar aromatic moieties, typically results in a high *T*_g_ and a strong tendency for aggregation.^[^ ^6^, ^7^ ^]^ As a result, many materials are brittle at room temperature and display a high tensile storage modulus *E*′ of up to several GPa.^[^ ^8^ ^]^ Synthetic efforts therefore concentrate on strategies that reduce *E*′, such as 1) copolymerization with a nonconjugated polymer, such as poly(methyl acrylate) or a polyurethane,^[^ ^9^, ^10^, ^11^ ^]^ 2) the incorporation of nonconjugated spacer units, such as hydrogen‐bonding motifs,^[^ ^12^, ^13^ ^]^ and flexible linkages,^[^ ^14^, ^15^, ^16^, ^17^, ^18^, ^19^, ^20^ ^]^ and 3) an increase in the length and grafting density of the solubilizing side chains.^[^ ^21^, ^22^ ^]^

The majority of conjugated polymers feature alkyl side chains, and an increase in their length is an effective way to lower the *T*_g_. Regio‐random poly(3‐alkylthiophene)s (P3AT)s, for instance, show a significant drop in *T*_g_ from +45 to −18 °C when increasing the alkyl side chain length from butyl to dodecyl,^[^ ^23^ ^]^ accompanied by a significant reduction in the shear storage modulus *G*′ at room temperature from 1 GPa to about 100 kPa.^[^ ^24^ ^]^ The decrease in storage modulus is less pronounced in case of regio‐regular P3ATs because the presence of crystallites reinforces the material above the *T*_g_, resulting in a much higher *E*′ ≈100 MPa at room temperature in case of poly(3‐dodecylthiophene) (P3DDT) with dodecyl side chains.^[^ ^21^, ^22^ ^]^

**Figure 1:**
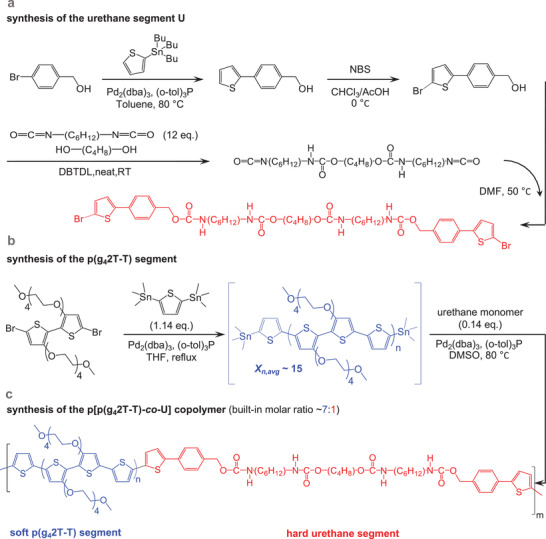
Synthesis scheme of a) the urethane segment (U), b) the p(g_4_2T‐T) segment, and c) the copolymer p[p(g_4_2T‐T)‐*co*‐U].

A different design strategy is required for conjugated polymers with oligoethylene glycol side chains, which currently receive widespread interest for applications related to bioelectronics,^[^ ^25^, ^26^, ^27^, ^28^ ^]^ as well as energy storage,^[^ ^29^, ^30^, ^31^ ^]^ and harvesting.^[^ ^32^, ^33^, ^34^, ^35^, ^36^ ^]^ These materials are considerably softer than comparable polymers with alkyl side chains. This behavior is exemplified by the polymer p(g_4_2T‐T) (see **Figure** **1** for chemical structure), which already softens at −45 °C (see discussion below) and displays a low tendency for aggregation.^[^ ^37^ ^]^

While the use of oligoethylene glycol side chains is an effective tool for the design of polymers with a low *T*_g_, the resulting material is too soft for applications, such as electronic textiles (e‐textiles) and thermoelectrics where mechanically robust and free‐standing structures are needed.^[^ ^38^, ^39^ ^]^ Therefore, it would be desirable to develop means to improve its mechanical robustness without unduly compromising its ability to transport and store electronic charges, facilitated by the conjugated backbone, as well as ions, the affinity for which is greatly enhanced by polar side chains.

The most widely used synthetic strategy to improve the ductility or elasticity of an initially rigid conjugated polymer involves the incorporation of a nonconjugated flexible spacer unit.^[^ ^14^, ^15^, ^16^, ^17^, ^18^ ^]^ We here invert this design principle and use a soft conjugated segment, i.e., p(g_4_2T‐T), which we combine with a hard linker unit. We chose to incorporate a urethane‐based block, which allows the formation of a reversible network mediated by hydrogen bonds. The resulting copolymer p[p(g_4_2T‐T)‐*co*‐U] (see Figure 1 for chemical structure) features a molar ratio of 7:1 of soft g_4_2T‐T to hard urethane segments. Incorporation of the urethane segments turns the putty‐like p(g_4_2T‐T) into a free‐standing solid with *E*′ ≈25 MPa. Incorporation of the hard urethane blocks only slightly reduces the electrical and electrochemical properties, yielding a material with a mobility of 0.2–0.7 cm^2^ V^−1^ s^−1^ when chemically doped or electrochemically oxidized, and a conductivity of about 20 S cm^−1^ when doped with 2,3,5,6‐tetrafluoro‐7,7,8,8‐tetracyanoquinodimethane (F4TCNQ).

## 2 Results and Discussion

### 2.1 Synthesis of the p[p(g_4_2T‐T)‐*co*‐U] Copolymer

The most widely used synthetic route to obtain segmented urethanes follows the reaction of an alcohol‐functionalized prepolymer with a diisocyanate and (subsequently) a diol or diamine chain extender.^[^ ^40^, ^41^ ^]^ Extending the same synthetic route to conjugated polymers seemed less reliable to us, as the typical synthesis scale (0.1–1 g) and large difference in molecular weight of the dihydroxyl‐functionalized conjugated macromonomer and the diisocyanate/chain extenders would prevent accurate control over the stoichiometry. To obtain a more robust synthetic procedure, we decided to first synthesize a high molecular‐weight monomer that incorporated the desired urethane bonds, allowing for a controlled one‐pot reaction with the conjugated soft block.

The reinforcing urethane monomer (Figure 1a) was obtained by first synthesizing (4‐(5‐bromothiophen‐2‐yl)phenyl)methanol as the capping moiety. Then, 1,4‐butanediol (BDO) was reacted with a 12‐fold excess of 1,6‐hexamethylene diisocyanate (HDI). After removal of excess HDI, the resulting HDI–BDO–HDI trimer was reacted with the bromothiophene‐functionalized benzyl alcohol to yield the reinforcing urethane monomer. The final monomer was moderately soluble in hot pyridine or hot dimethyl sulfoxide (DMSO).

To obtain the p[p(g_4_2T‐T)‐*co*‐U] copolymer, we first synthesized a p(g_4_2T‐T) prepolymer (Figure 1b) by reacting a ≈1.14 stoichiometric excess of 2,5‐bis(trimethylstannyl)thiophene with the dibrominated g_4_2T‐monomer under Stille conditions, which according to Carothers’ equation yields an average degree of polymerization of about ${\bar{X}}_{n}$ ≈15 in the limit of complete conversion, which corresponds to twice the average number of repeat units, i.e., 7 g_4_2T‐T + one thiophene ring and a concomitant *M*_n_ ≈ 4690 g mol^−1^. After reacting for 3 h, the stoichiometric imbalance was restored to a 1:1 stoichiometry by addition of 0.14 eq. of the urethane comonomer to the distannylated p(g_4_2T‐T) prepolymer. To ensure solubility of the final p[p(g_4_2T‐T)‐*co*‐U] copolymer during the chain‐extension, a solvent exchange was performed by addition of DMSO, while tetrahydrofuran (THF) was slowly removed through nitrogen purging and increasing the reaction temperature to 80 °C. The polymerization was continued for 48 h, after which the crude polymer was collected by precipitation. After purification, the Soxhlet extraction offered two fractions of p[p(g_4_2T‐T)‐*co*‐U]. A lower molecular weight fraction was obtained by dichloromethane (DCM) extraction, while a higher molecular weight fraction with a number‐average molecular weight of *M*_n_ ≈13.5 kg mol^−1^ (PDI ≈2.5, Figure S1, Supporting Information) was extracted with DMSO as a blue material, which is used in this study. From this value, and the weight of the built‐in urethane moiety (≈805 g mol^−1^), we can calculate that on average 3–6 reinforcing units are built into a polymer chain. ^1^H‐NMR indicated a ≈7:1 built‐in molar ratio of the g_4_2T‐T repeat unit and the urethane block (Figures 1c; and Figure S2, Supporting Information), i.e., a 6:1 weight ratio of g_4_2T‐T and the urethane block.

p(g_4_2T‐T) readily dissolves in chloroform (CH_3_Cl). Instead, the copolymer can be processed from 80 °C hot polar solvents, such as pyridine, DMSO and dimethylformamide. We used UV–vis spectroscopy to assess the stability of p[p(g_4_2T‐T)‐*co*‐U] dissolved in pyridine and DMSO. A blueshift of the polymer absorption occurs already after 1 day (Figure S3, Supporting Information), which indicates that solutions of the copolymer must be used immediately to avoid degradation. The homopolymer also displays limited stability in polar solvents (Figure S3, Supporting Information), as reported previously.^[^ ^37^ ^]^ Unlike solutions, thin films of both the homopolymer and copolymer, processed from pyridine, are stable for at least 3 months, as evidenced by the absence of any significant shift of the polymer absorption (Figure S4, Supporting Information). We however note that the polymer films become slightly oxidized when stored at ambient conditions, which we infer from the emergence of clear polaronic absorption peaks in the NIR part of the UV‐Vis‐NIR absorbance spectrum.

### 2.2 Hydrogen Bonding and Nanostructure of the Copolymer

**Figure 2:**
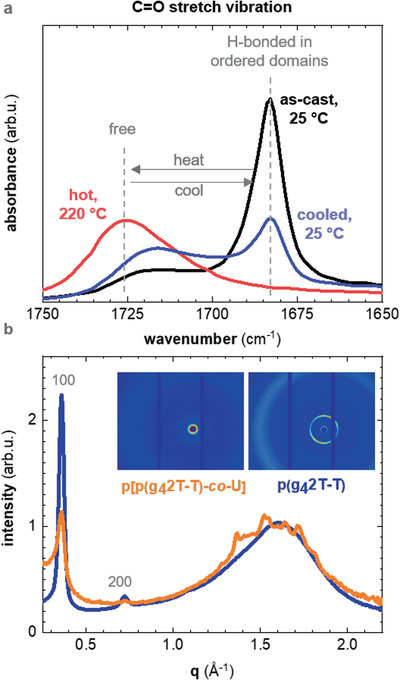
a) FTIR absorbance of the carbonyl stretch vibration in the urethane linkage, recorded at 25 °C for a p[p(g_4_2T‐T)‐*co*‐U] film cast from dimethyl sulfoxide (DMSO) (black), recorded for the same film at 220 °C (red) and after cooling from 220 to 25 °C (blue). b) WAXS diffractograms of a piece of p(g_4_2T‐T) (orange) and a free‐standing film of p[p(g_4_2T‐T)‐*co*‐U] (blue) normalized to the intensity of the amorphous halo at 1.6 Å^−1^.

In a first set of experiments, we studied how temperature impacts the formation of urethane “hard” domains in p[p(g_4_2T‐T)‐*co*‐U]. Transmission Fourier‐transform infrared (FTIR) spectroscopy allowed us to distinguish between free and hydrogen‐bonded urethane (**Figure** **2**a). Hydrogen bonding, depending on its strength, shifts the stretching vibration of NH and C=O groups of urethane bonds to lower energies.^[^ ^40^ ^]^ Chittibabu et al. studied a polythiophene with urethane containing side chains and assigned absorbance peaks at 1725 and 1705 cm^−1^ to the stretching vibration of free and hydrogen‐bonded C=O groups, respectively.^[^ ^42^ ^]^ p[p(g_4_2T‐T)‐*co*‐U] processed from DMSO features a pronounced peak at 1683 cm^−1^, which we assign to hydrogen‐bonded urethane segments, and a weaker shoulder around 1718 cm^−1^, which indicates free, i.e., not hydrogen‐bonded urethane segments. Heating to 220 °C results in the disappearance of the peak at 1683 cm^−1^ and a shift of the C=O stretching vibration to 1726 cm^−1^, which we explain with dissociation of hydrogen bonds. Upon subsequent cooling to room temperature the two C=O peaks at 1683 and 1718 cm^−1^ do not regain their initial intensity, which implies that the hydrogen bonded network only partially recovers. For the N—H stretch vibration (3324 cm^−1^), which we assign to the N—H stretch vibration of hydrogen‐bonded N—H groups, we observe a similar behavior (Figure S5, Supporting Information). This peak diminishes upon heating while a new peak at 3434 cm^−1^ emerges, indicating dissociation of hydrogen bonds. The peak at 3324 cm^−1^ only returns to some extent upon subsequent cooling to room temperature, which again confirms that hydrogen‐bonds only partially recover.

We carried out wide‐angle X‐ray scattering (WAXS) to compare the relative degree of crystalline order of p(g_4_2T‐T) and the p[p(g_4_2T‐T)‐*co*‐U] copolymer (Figure 2b). The WAXS diffractogram of neat p(g_4_2T‐T) features prominent peaks at *q*_100_ ≈0.36 Å^−1^ and *q*_200_ ≈ 0.71 Å^−1^, which we assign to lamellar stacking. We note that a distinct *π*–*π* stacking peak is absent, in agreement with previous reports.^[^ ^37^, ^43^ ^]^ Instead, a broad amorphous halo with a peak maximum at *q* ≈1.6 Å^−1^ is present. For the copolymer the intensity of the lamellar stacking peaks *q*_*n*00_, assigned to the p(g_4_2T‐T) blocks, is noticeably reduced. We argue that the presence of the urethane blocks reduces the ability of the conjugated segment to order due to formation of the hydrogen‐bonded network. Further, the WAXS diffractogram of the copolymer features weak but distinct peaks on top of the broad amorphous halo. We rule out that some of these peaks arise from *π*–*π* stacking of p(g_4_2T‐T), which should result in a peak around 1.8 nm^−1^ (cf. refs. ^[^ ^37^, ^43^ ^]^). Instead, we tentatively assign these additional peaks to the presence of ordered urethane domains. The length of a fully extended urethane block in our copolymer is about 5 nm, and therefore ordered domains are likely only a few nanometers in size. We attempted to resolve the nanostructure of neat p(g_4_2T‐T) as well as the copolymer with high‐resolution scanning transmission electron microscopy (HR‐STEM) but did not observe distinct domains (Figure S6, Supporting Information). A possible explanation for this is that the domains are evenly distributed in three dimensions and cannot be distinguished in projection from a sample where multiple blocks are overlapping. Both materials display the same granular texture, which may form as a result of the micellar‐like structure that we have previously inferred for solutions of neat p(g_4_2T‐T).^[^ ^37^ ^]^

### 2.3 Thermomechanical Properties of the Copolymer and Comparison with p(g_4_2T‐T)

Since p(g_4_2T‐T) is very soft, we were unable to study the thermomechanical properties of free‐standing samples of the reference material. We instead used a technique recently applied to conjugated polymers by Sharma et al.,^[^ ^44^ ^]^ which utilizes a thin layer of the polymer supported by a glass fiber mesh and allows to record the relative change in tensile storage and loss modulus *E*′ and *E*″ upon heating. A dynamic mechanical analysis (DMA) thermogram of fiber mesh reinforced p(g_4_2T‐T) indicates considerable softening already at low temperatures with a peak in *E*″ at −45 °C and a shoulder around −20 °C (Figure S7, Supporting Information). For the copolymer we observe two peaks in *E*″ at −44 and −20 °C. While these measurements clearly indicate that both materials start to soften at very low temperatures, we are currently unable to conclusively assign either observed transition to the *T*_g_. We have used the empirical relation proposed by Xie et al.,^[^ ^24^ ^]^ which allows to estimate the *T*_g_ of a conjugated polymer by considering the mobility of each atom that make up its repeat unit, and obtain a value of *T*_g_ = −15 °C (cf. Supporting Information).

**Figure 3:**
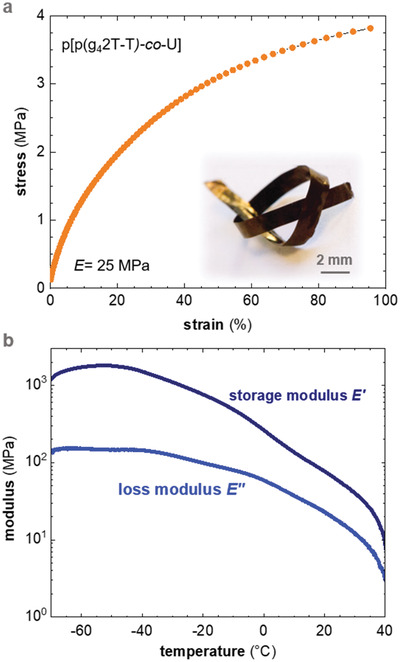
a) Tensile stress–strain curve of a free‐standing p[p(g_4_2T‐T)‐*co*‐U] film (inset: photograph of a knotted p[p(g_4_2T‐T)‐*co*‐U] ribbon). b) Storage and loss modulus, *E*′ and *E*″, of free‐standing p[p(g_4_2T‐T)‐*co*‐U] measured with DMA from −70 to 40 °C.

We were readily able to prepare micrometer‐thick, free‐standing samples from the copolymer by peeling off drop‐cast films from a glass substrate. The copolymer is considerably stronger compared to neat p(g_4_2T‐T), as evidenced by a knot tied into a ribbon cut from a drop‐cast copolymer film (**Figure** **3**a). We studied the thermomechanical properties of the copolymer both with tensile testing and DMA. The tensile storage modulus of the copolymer has a value of *E* ≈25 MPa at room temperature (value from single measurement shown in Figure 3a). A strain at break of *ε*_break_ ≈95% indicates that the copolymer is able to undergo significant plastic deformation. DMA measured in tensile mode revealed that *E*′ drops from 1.2 GPa at −70 °C to 7 MPa at 40 °C (Figure 3b).

### 2.4 Electrochemical Swelling

**Figure 4:**
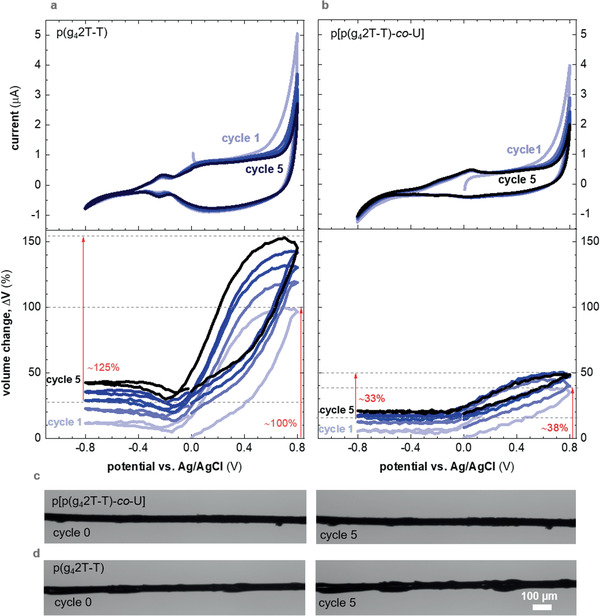
Cyclic voltammograms (top) and volume change of the polymer coating $\Delta V\;=\;\left(V_{\mathrm{pol}}-V_{\mathrm{pol}}^{0}\right)/V_{\mathrm{pol}}^{0}$, where *V*_pol_ is the volume of the coating at different potentials and $V_{\mathrm{pol}}^{0}$ is the initial volume of the coating (bottom), recorded during oxidation/reduction cycle 1–5 of a carbon filament coated with ~2 µm of a) p(g_4_2T‐T) and b) p[p(g_4_2T‐T)‐*co*‐U] in 0.01 m KCl electrolyte; c,d) optical micrographs of the coated carbon filaments prior to the first cycle and after cycle 5.

We used swelling experiments to confirm the presence of a reinforcing network in the p[p(g_4_2T‐T)‐*co*‐U] copolymer. Recent studies have shown that polythiophenes with oligoethylene glycol side chains undergo considerable active swelling exceeding 100% during electrochemical oxidation.^[^ ^28^, ^45^ ^]^ Our setup consisted of a carbon filament working electrode coated with a ≈2 µm thick layer of the homopolymer or the copolymer, which was submerged in an aqueous KCl electrolyte solution together with a Pt counter electrode and an Ag/AgCl reference electrode (cf. Experimental Section for details). Application of a positive potential of up to +0.8 V versus Ag/AgCl resulted in oxidation of the polymer layer, accompanied by ingression of Cl^−^ counterions that are surrounded by a water hydration shell, which leads to a sizable volume increase. We monitored the degree of swelling as a function of oxidation/reduction cycle by applying a potential of ±0.8 V versus Ag/AgCl. Very little passive swelling, less than 1%, is observed for both polymers (Figure S8, Supporting Information). For neat p(g_4_2T‐T) a relative volume change of Δ*V* ≈100% (i.e., the volume change upon swelling with respect to the previous minimum contracted state) increased to Δ*V* ≈125% during the 5th electrochemical cycle. Instead, the ability of the p[p(g_4_2T‐T)‐*co*‐U] copolymer to expand is lower with only Δ*V* ≈38% during the 1st electrochemical cycle and Δ*V* ≈33% during the 5th cycle (**Figure** **4**). Both polymers exhibit an irreversible volume change, i.e., they do not return to the initial state when electrochemically reduced. The irreversible change tends to stabilize at the 5th cycle for p(g_4_2T‐T), while the copolymer stabilizes already at the 3rd cycle. Such a behavior has been observed before for similar materials like p(g_3_T2)^[^ ^45^ ^]^ as well as for hydrogels, where it has been described as a conditioning effect due to nonrecoverable changes in the polymer network.^[^ ^46^ ^]^ We argue that the presence of urethane domains allows the copolymer to resist extensive swelling and renders the polymer matrix more stable, which is in agreement with the presence of a reinforcing network that we inferred from our thermomechanical analysis.

### 2.5 Electrochemical Oxidation and Spectroelectrochemistry

**Figure 5:**
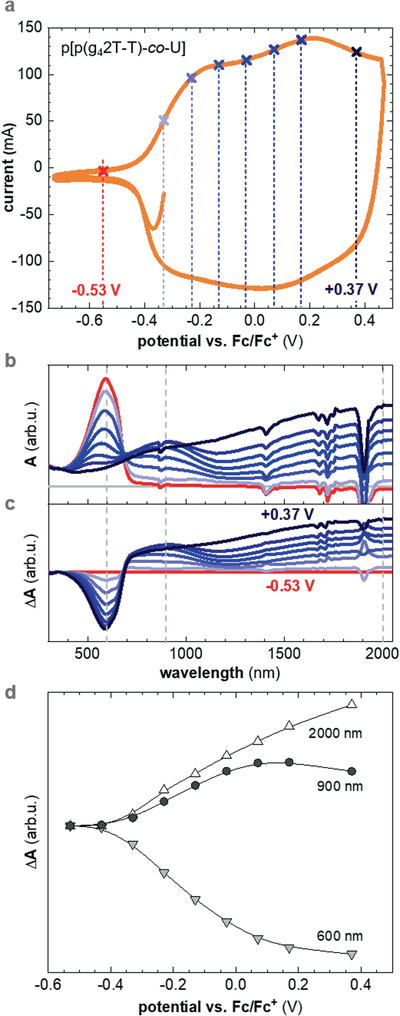
a) Cyclic voltammogram of p[p(g_4_2T‐T)‐*co*‐U] film in 0.1 m solution of [EMIM][BF_4_]. b) UV–vis–NIR absorbance spectra recorded at each applied electrochemical potential, and c) difference in absorbance Δ*A* between neutral and oxidized thin films. d) Δ*A* at 600 nm, 900 nm, and 2000 nm versus oxidation potential.

We were interested in comparing the extent to which p(g_4_2T‐T) and the p[p(g_4_2T‐T)‐*co*‐U] copolymer can be electrochemically oxidized. Our electrochemical cell contained a polymer film spin‐cast on an indium tin oxide (ITO)/glass working electrode, a Pt wire counter electrode and an Ag wire pseudoreference electrode, submerged in an electrolyte solution of 0.1 m 1‐ethyl‐3‐methylimidazolium tetrafluoroborate ([EMIM][BF_4_]) in acetonitrile (AcN; cf. Experimental Section for details). Cyclic voltammograms of the homopolymer and the copolymer indicate an oxidation onset of *E*_ox_ ≈ −0.44 V and −0.41 V versus Ferrocene/Ferrocenium (Fc/Fc^+^) (**Table** **1** and **Figure** **5**a), which correspond to an ionization energy IE = 5.1 eV + *E*_ox_ ≈ 4.66 V and 4.69 eV, respectively.

**Table 1:** Summary of electrochemical oxidation and chemical doping experiments: degree of active swelling Δ*V* (*n* = 1), conductivity *σ* when sequentially doped with F4TCNQ (*n* = 3), charge‐carrier density *N*_v_ estimated from UV–vis–NIR absorbance spectra of F4TCNQ doped thin films (*n* = 1), and corresponding charge‐carrier mobility *μ*; number of measured samples, *n*, indicated in brackets

	p(g_4_2T‐T)	p[p(g_4_2T‐T)‐*co*‐U]
Δ*V* [%]	125	33
*σ* [S cm^−1^]	48 ± 8	20 ± 5
*N*_v_ [m^−3^]	2.4 × 10^26^	1.7 × 10^26^
*μ* [cm^2^ V^−1^ s^−1^]	1.2 ± 0.2	0.7 ± 0.2

We then recorded a series of UV–vis–NIR absorbance spectra at different constant oxidation potentials between −0.33 V and +0.37 V versus Fc/Fc^+^ and plotted the change in absorbance relative to the spectrum of the undoped polymer at −0.53 V (Figure 5b). The neutral p(g_4_2T‐T) absorption with its peak at 600 nm diminishes with increasing potential due to gradual oxidation of the conjugated backbone. At +0.37 V the change in absorbance Δ*A* at 600 nm has reached a close to constant value, indicating that both polymers are strongly oxidized. We conclude that the urethane segments do not noticeably impact the ability of the copolymer to take up charge. At higher wavelengths two polaronic absorption bands emerge, one with its peak at 900 nm and one in the infrared region. The absorbance at 900 nm increases up to an oxidation potential of 0.07 V, but decreases again at higher potentials, which we explain with the increasing presence of bipolarons (Figure 5c; and Figure S9, Supporting Information). We argue that at low oxidation potentials the majority of hole charges are polarons, and that bipolarons increasingly form at higher oxidation levels.

### 2.6 Chemical Doping with F4TCNQ

Both p(g_4_2T‐T) and p[p(g_4_2T‐T)‐*co*‐U] could be readily doped with F4TCNQ. Sequential doping by drop‐coating ≈60 nm thin polymer films with solutions of F4TCNQ in AcN resulted in an electrical conductivity of *σ* ≈ (20 ± 5) S cm^−1^ and (48 ± 8) S cm^−1^ for p[p(g_4_2T‐T)‐*co*‐U] and p(g_4_2T‐T), respectively. The conductivity of the doped homopolymer and copolymer gradually decreased over the course of 7 days accompanied by a decrease in the polaronic absorption peaks in the NIR (Figure S11, Supporting Information).

**Figure 6:**
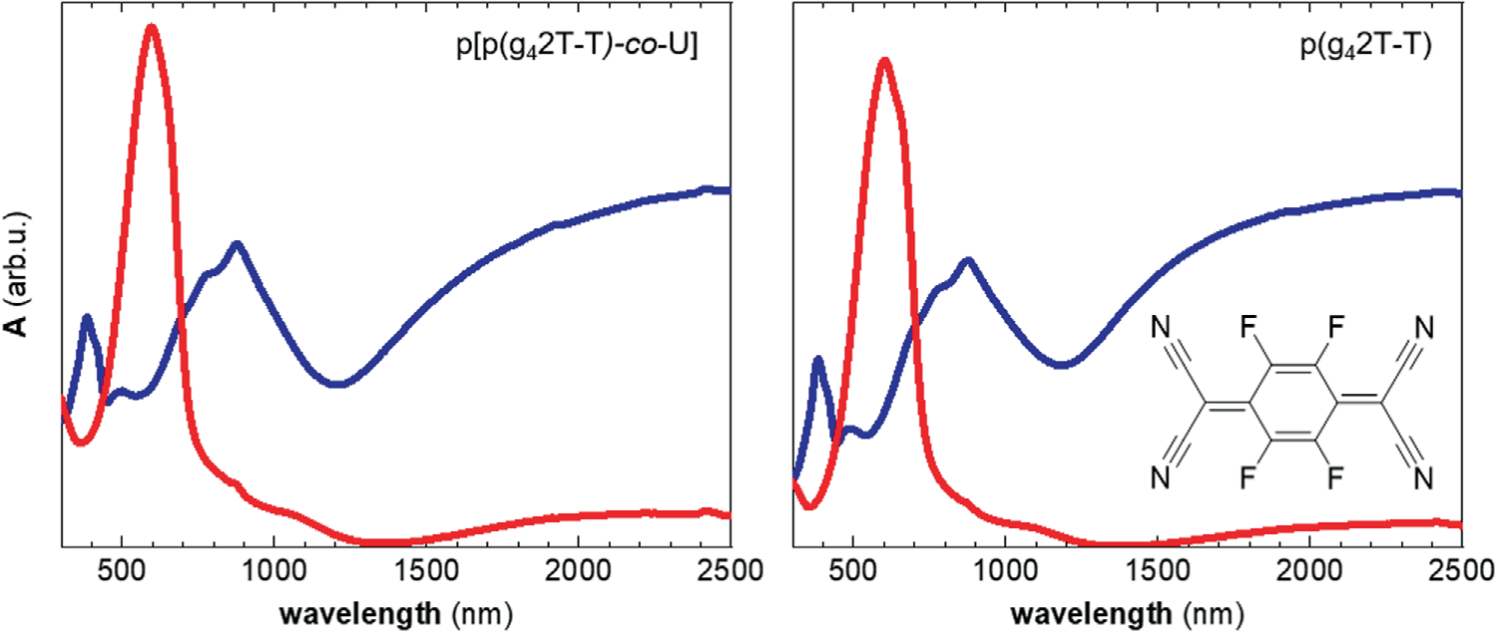
UV–vis–NIR absorbance spectra of ≈60 nm thin films of the copolymer p[p(g_4_2T‐T)‐*co*‐U] (left) and p(g_4_2T‐T) (right) after spin‐coating (red) and once sequentially doped with F4TCNQ (blue); inset: chemical structure of F4TCNQ.

We recorded UV–vis–NIR absorbance spectra of F4TCNQ‐doped thin films. In case of both polymers the neat p(g_4_2T‐T) absorption peak at 600 nm disappears, while two polaronic absorbance bands emerge around 900 nm and in the infrared, respectively (**Figure** **6**). We estimated the F4TCNQ anion concentration through comparison of the UV–vis–NIR absorbance spectra of the F4TCNQ‐doped polymers with those of neat F4TCNQ and the F4TCNQ anion (Figure 6; and Figure S10, Supporting Information), as described previously.^[^ ^37^ ^]^ We estimate a concentration of about 10^26^ anions per m^−3^ for both materials (Table 1). The F4TCNQ anion concentration is equal to the number of generated hole polarons and hence corresponds to the charge‐carrier density *N*_v_, including both bound and mobile charges. We estimate the charge‐carrier mobility *μ* according to *σ*  = *N*_v_  · *μ* · *e* where *e* is the elementary charge, and obtain a value of *μ* ≈ (1.2 ± 0.2) cm^2^ V^−1^ s^−1^ for p(g_4_2T‐T) and *μ* ≈ (0.7 ± 0.2) cm^2^ V^−1^ s^−1^ for p[p(g_4_2T‐T)‐*co*‐U] (Table 1). A comparison of the mobility values indicates that the introduction of urethane blocks, which leads to a reduction in the ordering of p(g_4_2T‐T) segments as a result of the formation of a hydrogen‐bonded network (see WAXS diffractograms, Figure 2b), only slightly reduces *μ*.

### 2.7 Organic Electrochemical Transistors (OECTs)

**Figure 7:**
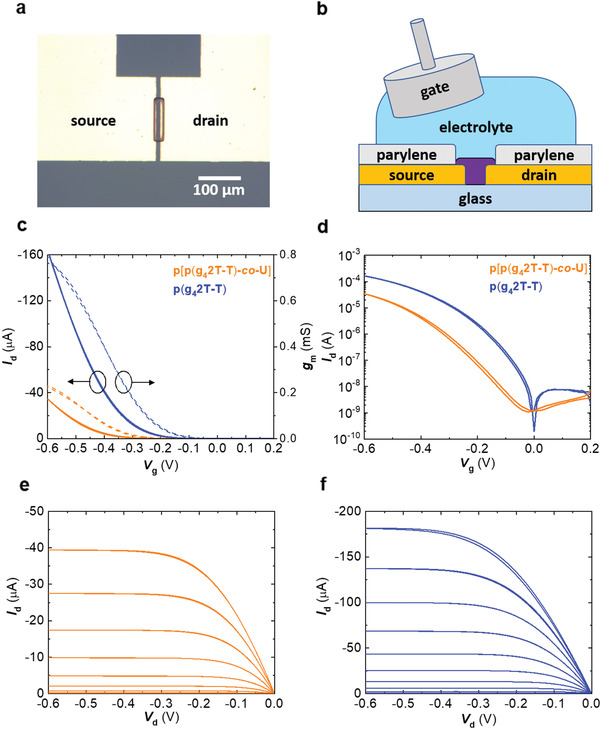
OECTs: a) Micrograph of the patterned semiconducting channel connecting the encapsulated Au source and drain electrodes; b) cross‐sectional cartoon of the OECT device structure (not to scale); c) transfer curves (*I*_d_ vs *V*_g_) and voltage dependent gate transconductance *g*_m_ of p[p(g_4_2T‐T)‐*co*‐U] (orange) and p(g_4_2T‐T) (blue) OECTs with a drain bias of −0.6 V. d) Semilog plots of the same transfer curves; output curves of e) p[p(g_4_2T‐T)‐*co*‐U] and f) p(g_4_2T‐T) OECTs. All measurements collected at a sweep rate of 200 mV s^−1^ on devices with ≈20 nm thick channels and a width to length ratio W/L = 10.

The mixed conducting properties of conjugated polymers with oligoethylene glycol side chains make them ideal candidates for OECTs.^[^ ^47^, ^48^ ^]^ Integration of OECTs onto flexible arrays for implantable or wearable bioelectronic applications requires mechanical robustness,^[^ ^5^, ^49^ ^]^ which urethane blocks readily impart. Therefore, OECTs were fabricated and tested employing the homopolymer and copolymer as the semiconducting channel material (**Figure** **7**a,b), with the various device parameters collected in **Table** **2**. Equally thin p(g_4_2T‐T) and p[p(g_4_2T‐T)‐*co*‐U] devices (thickness *d* ≈ 20 nm) with identical channel aspect ratio (width to length ratio W/L = 10) both displayed clear transistor behavior (Figure 7c) with a strong turn on (subthreshold swing of (75 ± 2) mV/decade and (87 ± 5) mV/decade, respectively), good ON/OFF ratios of more than 10^4^ (Figure 7d), and ideal saturation (Figure 7e,f). The volumetric capacitance C* for both materials was determined by electrochemical impedance spectroscopy (EIS; Figure S12, Supporting Information). The incorporation of the urethane block had little effect on the volumetric capacitance, with values of C* ≈ (258 ± 102) F cm^−3^ for p(g_4_2T‐T) and C* ≈ (279 ± 114) F cm^−3^, for p[p(g_4_2T‐T)‐*co*‐U], which are in good agreement with measurements done on other polythiophenes with oligoethylene glycol side chains.^[^ ^50^ ^]^ From the slope of the collected transfer curves (*I*_d_ vs *V*_g_), the gate transconductance *g*_m_ =  d*I*_d_/d*V*_g_ was calculated, which captures the ability of an OECT to amplify an input. In the saturation regime *g*_m_ is given by

(1) $$\begin{array}{c} g_{m}=\frac{Wd}{L}\;\mu C^{*}\left(V_{T}-V_{G}\right) \end{array}$$

where µC^*^, the product of volumetric capacitance and charge‐carrier mobility *µ*, represents a figure of merit intrinsic to the channel material. Given the channel dimensions, and p(g_4_2T‐T) and p[p(g_4_2T‐T)‐*co*‐U] threshold voltages of *V*_t_ ≈ (−202 ± 5) mV and (−314 ± 5) mV, respectively, μC* was directly extractable from the transfer curves. The polymers p(g_4_2T‐T) and p[p(g_4_2T‐T)‐*co*‐U] displayed a μC* ≈ (86 ± 39) F cm^−1^ V^−1^ s^−1^ and (36 ± 29) F cm^−1^ V^−1^ s^−1^, respectively. We ascribe the 60% decrease in μC* to incorporation of urethane blocks, which led to a decrease in *μ* (cf. Table 2). The charge‐carrier mobility in the saturation regime *μ*_sat_ was extracted from the slope of $\sqrt{I_{\mathrm{sd}}}$ versus *V*_g_ plots. As expected, *μ*_sat_ of p[p(g_4_2T‐T)‐*co*‐U] was ≈60% less than that of p(g_4_2T‐T) (Table 2). Alternatively, calculating the figure of merit from the product of transfer curve determined mobility and EIS determined capacitance (*μ*_sat_ C*) gave similar values and an identical trend. The μC* and *μ*_sat_ C* trends for OECTs both mirror the mobility and conductivity trends observed in molecularly doped samples.

**Table 2:** OECT parameters and material figures of merit (*n* = 6): thickness *d*, threshold voltage *V*_t_ and saturation mobility *μ*_sat_ extracted from fits of $\sqrt{I_{d}}$ versus *V*_g_ plots; average volumetric capacitance C* beyond the threshold voltage determined by electrochemical impedance spectroscopy (EIS; *n* = 2); maximum transconductance *g*_m_ and μC* extracted from the slope of saturated transfer curves at *V*_g_ = −0.6 V; number of measured samples, *n*, indicated in brackets

	p(g_4_2T‐T)	p[p(g_4_2T‐T)‐*co*‐U]
*d* [nm]	22 ± 9	18 ± 10
*V*_t_ [mV]	−202 ± 5	−314 ± 5
*μ*_sat_ [cm^2^ V^−1^ s^−1^]	0.39 ± 0.07	0.15 ± 0.10
C* [F cm^−3^]	258 ± 102	279 ± 114
μC* (F cm^−1^ V^−1^ s^−1^)	86 ± 39	36 ± 29
*μ*_sat_ C* [F cm^−1^ V^−1^ s^−1^]	100 ± 44	42 ± 29
*g*_m_ [mS]	0.73 ± 0.14	0.18 ± 0.10

Interestingly, C* is similar for both the homopolymer and copolymer despite a smaller volume fraction of conjugated repeat units in case of the former, which indicates that the presence of urethane blocks actually leads to a higher oxidation level of the conjugate backbone. While the presence of urethane blocks does not inhibit the overall charge storage capacity, C*, it does delay the onset voltage of charge accumulation *V*_t_. The incorporation of 5 nm long nonconjugated segments that significantly disrupt the ability of conjugated segments to order (cf. Figure 2) likely impairs electronic charge transport. However, the copolymers resistance to swelling seems to counterbalance this, as electrically conductive pathways clearly persist as evidenced by the OECT results.

## 3 Conclusions

We have synthesized a copolymer consisting of soft p(g_4_2T‐T) segments and hard urethane segments. The urethane segments considerably reinforce the polar polythiophene through hydrogen bonding. Free‐standing ribbons of the p[p(g_4_2T‐T)‐*co*‐U] copolymer feature a tensile modulus of about 25 MPa and elongation at break of 95%. The presence of the reinforcing urethane segments does not affect the ability to take up charge upon electrochemical oxidation. Both, chemical doping with F4TCNQ and the operation of OECTs indicate that the urethane block only slightly reduces the charge‐carrier mobility. We conclude that the introduction of reinforcing segments is a promising strategy for modifying the mechanical and electrochemical properties of polar conjugated polymers. Further work with regard to the type and relative length of the reinforcing segments is needed to create materials that do not show any trade‐off between mechanical and electrochemical properties.

## 4 Experimental Section

##### Materials

The synthesis of p(g_4_2T‐T) (*M*_n_ ≈ 24 kg mol^−1^; PDI ≈ 3.3) is described elsewhere.^[^ ^37^ ^]^ The synthetic procedure and corresponding NMR spectra of p[p(g_4_2T‐T)‐*co*‐U] (*M*_n_ ≈ 13.5 kg mol^−1^; PDI ≈ 2.5) are provided in the Supporting Information. Chloroform (Fisher Scientific), acetonitrile (AcN) (Fisher Scientific), 1,2‐ dichlorobenzene (o‐DCB) (Acros Organics), and anhydrous pyridine (Sigma‐Aldrich) where used as received. DMSO from Fisher Scientific was dry distilled and stored over 4 Å molsieves. 2,3,5,6‐tetrafluoro‐7,7,8,8‐tetracyanoquinodimethane (F4TCNQ) and 1‐ethyl‐3‐methylimidazolium tetrafluoroborate ([EMIM][BF_4_]) were purchased from Tokyo Chemical Industry (TCI) and Sigma‐Aldrich, respectively, and used as received.

##### Chemical Doping

Films for UV–vis–NIR and conductivity measurements were prepared by spin‐coating 80–90 °C hot solutions of the polymers in anhydrous pyridine (10 g L^−1^) onto hot glass slides. Sequential doping was done at room temperature by drop‐casting a solution of F4TCNQ in AcN (10 g L^−1^) onto thin films, followed by spinning off the remaining solution after 1 min. Doped films were rinsed with AcN to remove excess dopant. The thickness of thin films was measured with a KLA Alphastep Tencor D‐100 profilometer.

##### Size Exclusion Chromatography (SEC)

The molecular weight distribution was measured at 40 °C with a TOSOH EcoSEC HLC‐8320GPC system (Japan), equipped with an EcoSEC RI detector and three PSS PFG 5 µm columns (microguard, 100, and 300 Å; USA). Poly(methyl methacrylate) (PMMA) standards were used for calibration and toluene was used as an internal standard.

##### NMR Spectroscopy

NMR spectra were recorded with an automated Agilent (Varian) MR 400 MHz spectrometer (equipped with “one‐probe”) with CDCl_3_ or d‐DMSO as the solvent. In all cases, the peak values were calibrated relative to the residual solvent signals (CDCl_3_, 7.26 ppm or d‐DMSO, 2.50 ppm).

##### FTIR

Transmission FTIR spectra were recorded with a PerkinElmer FT‐IR Spectrometer “Frontier” on p[p(g_4_2T‐T)‐*co*‐U] drop‐cast from DMSO (10 g L^−1^) onto CaF_2_. Variable‐temperature transmission FTIR was done by heating from 22 to 220 °C using a Specac electrical heating jacket equipped with a Specac 4000 series temperature controller (West 6100^+^).

##### UV–Vis–NIR Absorption Spectroscopy

UV–vis–NIR spectra were recorded with a PerkinElmer Lambda 1050 spectrophotometer.

##### Analysis of Mechanical Properties

Free‐standing films of p[p(g_4_2T‐T)‐*co*‐U] with a thickness of ≈100 µm were prepared by drop‐casting from pyridine (10 g L^−1^) onto microscopy glass slides, from which the specified film could be removed with a sharp blade. Samples of thin films supported by a glass fiber mesh were prepared by coating glass fiber meshes (50 × 5 mm^2^) with solutions of the polymers dissolved in pyridine (15 g L^−1^). DMA and tensile testing were performed using a Q800 (TA Instruments); glass fiber mesh samples were clamped with the glass fiber strands at 45° to the direction of deformation. DMA was carried out at a dynamic strain of 0.05% and a frequency of 1 Hz while ramping the temperature from −90 to 140 °C (glass fiber mesh samples) and −70 to 40 °C (free‐standing p[p(g_4_2T‐T)‐*co*‐U], gauge length = 4.5 mm) at 3 °C min^−1^ with a preload force of 0.01 N. Tensile testing was performed at room temperature and a strain rate of 0.5 N min^−1^ with a preload force of 0.02 N and gauge length = 3.8 mm.

##### WAXS

WAXS was done using a piece of as‐synthesized p(g_4_2T‐T) and a film sample prepared for DMA in case of p[p(g_4_2T‐T)‐*co*‐U]. WAXS diffractograms were obtained using a Mat:Nordic instrument from SAXSLAB equipped with a Rigaku 003+ high brilliance micro focus Cu‐radiation source (wavelength = 1.5406 Å) and a Pilatus 300 K detector placed at a distance of 88.6 mm from the sample.

##### Transmission Electron Microscopy (TEM)

Samples for TEM were prepared by spin‐coating p(g_4_2T‐T) from chloroform (1 g L^−1^) or p[p(g_4_2T‐T)‐*co*‐U] from anhydrous pyridine (1 g L^−1^) onto glass slides coated with poly(diallyldimethylammonium chloride) (PDADMAC). Pieces of polymer films were floated off in water and collected with a copper grid. TEM was done with a FEI Titan 80–300 operated in STEM mode at an acceleration voltage of 300 kV. Images were recorded using a signal from a high‐angle annular dark‐field detector.

##### Spectroelectrochemistry

Electrochemical measurements were performed with freshly prepared solutions of [EMIM][BF_4_] in dry and degassed AcN (0.1 m) using a custom made three‐electrode setup in a standard 1  × 1 cm^2^ quartz cuvette. Polymer films were spin‐coated from pyridine (10 g L^−1^) onto ITO coated glass (R ≈150 Ohm sq^−1^), which served as the working electrode. A Pt wire (∅ ≈ 1 mm) and Ag wire served as the counter and pseudoreference electrode. The potentials were calibrated versus the Ferrocene/Ferrocenium (Fc/Fc^+^) redox couple. Cyclic voltammograms were recorded with a scan rate of 100 mV s^−1^ using a 650D electrochemical workstation from CH Instruments. The ionization energies of the polymers were calculated using IE = 5.1 eV + *E*_ox_ versus Fc/Fc^+^, where *E*_ox_ is the oxidation onset versus Fc/Fc^+^. Spectroelectrochemistry was performed by recording UV–vis–NIR spectra at different oxidation potentials with a PerkinElmer Lambda 1050 spectrophotometer.

##### Swelling Experiments

Carbon filaments (diameter ≈ 34.5 µm ± 2.5 µm; provided by Specialty Materials, USA) were coaxially coated with p(g_4_2T‐T) from chloroform or p[p(g_4_2T‐T)‐*co*‐U] from pyridine, and left to dry at room temperature. The electrochemical cell consisted of a coated carbon filament working electrode (and reference uncoated fiber), a Pt counter electrode and an Ag/AgCl wire reference electrode, which were arranged between two glass slides separated by a poly(dimethylsiloxane) (PDMS) well that contained the electrolyte (0.01 m KCl). Cyclic voltammetry was performed with a Metrohm μAutolab Type III (NOVA 2.1 software) between ± 0.8 V at a scan rate of 10 mV s^−1^, with simultaneous monitoring of the volume change using a Nikon SMZ1500 stereo microscope equipped with a Nikon DS‐Fi1 camera. The volume of the swollen polymer coating at different potentials was calculated from the average width of the coated carbon filament assuming a cylindrical shape.

##### OECTs

OECTs test chips were prepared following previously reported microfabrication techniques.^[^ ^51^ ^]^ OECT channels were fabricated by spin‐coating p(g_4_2T‐T) from chloroform (2.5 g L^−1^) or p[p(g_4_2T‐T)‐*co*‐U] from filtered o‐DCB solution (2 g L^−1^) onto OECT test chips at room temperature, followed by patterning via removal of a sacrificial parylene layer. OECTs were gated with aqueous 100 × 10^−3^ m NaCl using a Ag/AgCl pellet as the faradaic gate electrode.^[^ ^52^ ^]^ Electrical characterization of the OECTs was carried out using source‐measure units from National Instruments controlled by custom LabView code. The capacitance was determined via EIS using a Metrohm potentiostat with a frequency response analyzer with a Ag/AgCl pellet functioning as a combined reference and counter electrode.^[^ ^52^ ^]^

##### Electrical Conductivity Measurements

The electrical resistivity was measured with a 4‐point probe setup from Jandel Engineering (cylindrical probe head, RM3000) using colinear tungsten carbide electrodes with equidistant spacing of 1 mm. The in‐line 4‐point probe for films gives a measure of the sheet resistance $r_{s}=\frac{\pi }{\ln2}\cdot V/I$, where *π*/ln 2 is a geometrical correction factor. The conductivity was calculated according to *σ*  =  1/(*t* · *r*_s_).

##### Statistical Analysis

The number of measured samples are given in the legends of Tables 1 and 2; reported values and errors correspond to the mean and standard deviation.

## Conflict of Interest

The authors declare no conflict of interest.

## Supporting information

Supporting Information
