# Supplementary material for: Toughening of a Soft Polar Polythiophene through Copolymerization with Hard Urethane Segments
Source: Adv Sci (Weinh). 2020 Dec 11;8(2):2002778. doi: 10.1002/advs.202002778 (PMC7816697; doi:10.1002/advs.202002778)
Supplement: Supplementary file 1 — Supporting Information [file ADVS-8-2002778-s001.pdf]

© 2020 Wiley-VCH GmbH

ADVANCED SCIENCE
Open Access

Supporting Information

for *Adv. Sci.*, DOI: 10.1002/advs.202002778

Toughening of a Soft Polar Polythiophene through Copolymerization with Hard Urethane Segments

*Sepideh Zokaei,[+] Renee Kroon,[+] Johannes Gladisch, Bryan D. Paulsen, Wonil Sohn, Anna I. Hofmann, Gustav Persson, Arne Stamm, Per-Olof Syrén, Eva Olsson, Jonathan Rivnay, Eleni Stavrinidou, Anja Lund, Christian Müller**

Supporting Information

Toughening of a Soft Polar Polythiophene through Copolymerization with Hard Urethane Segments

Sepideh Zokaei,[1+] Renee Kroon,[1+] Johannes Gladisch,[2,3] Bryan D. Paulsen,[4] Wonil Sohn,[4] Anna I. Hofmann,[1] Gustav Persson,[5] Arne Stamm,[6] Per-Olof Syrén,[6,7] Eva Olsson,[5,8] Jonathan Rivnay,[4] Eleni Stavrinidou,[2,3] Anja Lund,[1] Christian Müller[1,8]*

[1] Department of Chemistry and Chemical Engineering, Chalmers University of Technology, 41296 Göteborg, Sweden

[2] Laboratory of Organic Electronics, Department of Science and Technology, Linköping University, 60174 Norrköping, Sweden

[3] Wallenberg Wood Science Center, Linköping University, Norrköping, Sweden

[4] Department of Biomedical Engineering, Northwestern University, Evanston, IL 60208, USA

[5] Department of Physics, Chalmers University of Technology, 41296 Göteborg, Sweden

[6] Department of Fibre and Polymer Technology, KTH Royal Institute of Technology, 11428 Stockholm, Sweden

[7] Wallenberg Wood Science Center, KTH Royal Institute of Technology, Stockholm, Sweden

[8] Wallenberg Wood Science Center, Chalmers University of Technology, Göteborg, Sweden

[+] equal contribution

* e-mail: christian.muller@chalmers.se

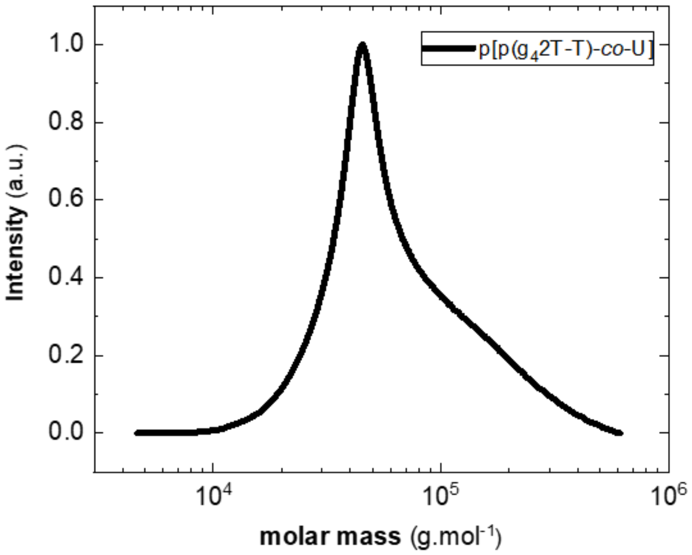

Figure S1. Molecular weight distribution of the larger molecular weight fraction of p[p(g42T-T)-*co*-U] from DMF.

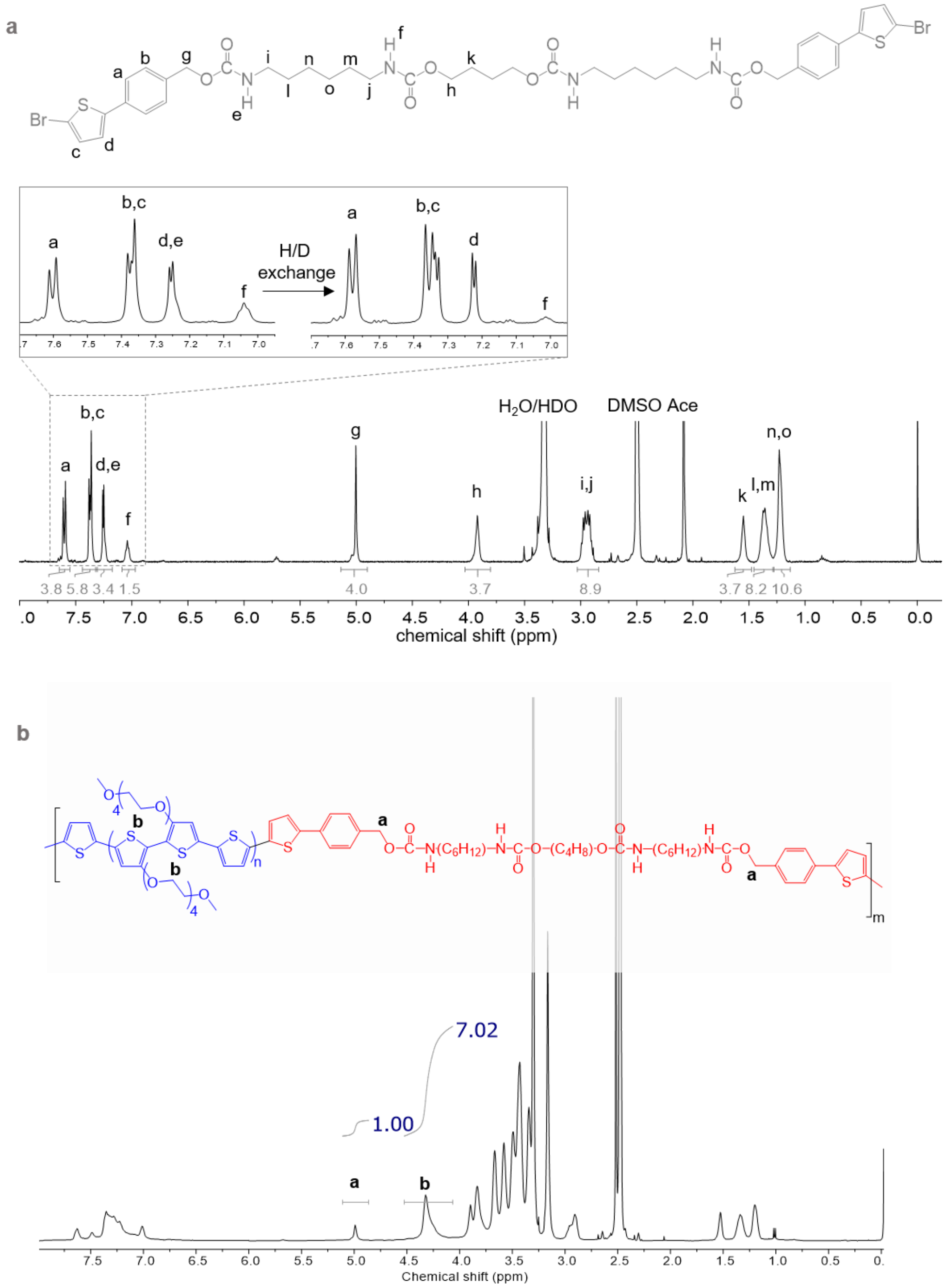

Figure S2. a) ^{1}H-NMR of the urethane segment (compound 4; cf. synthetic procedures). The overlap of N-H proton e with proton d can be shown by performing a H-D exchange, removing the contribution of the N-H peak to the multiplet and restoring the symmetry of the proton d peak; (b) ^{1}H-NMR of p[p(g$_4$2T-T)-*co*-U]. The Ph-<u>CH$_2$</u>-O-R peak (5.01 ppm) and the thiophene-O-<u>CH$_2$</u>-R (4.3 ppm) correspond to the urethane monomer and p(g$_4$2T-T) prepolymer, respectively. These peaks yield a built-in molar ratio of 7:1 between the g$_4$2T-T repeat unit and the urethane block in the final polymer.

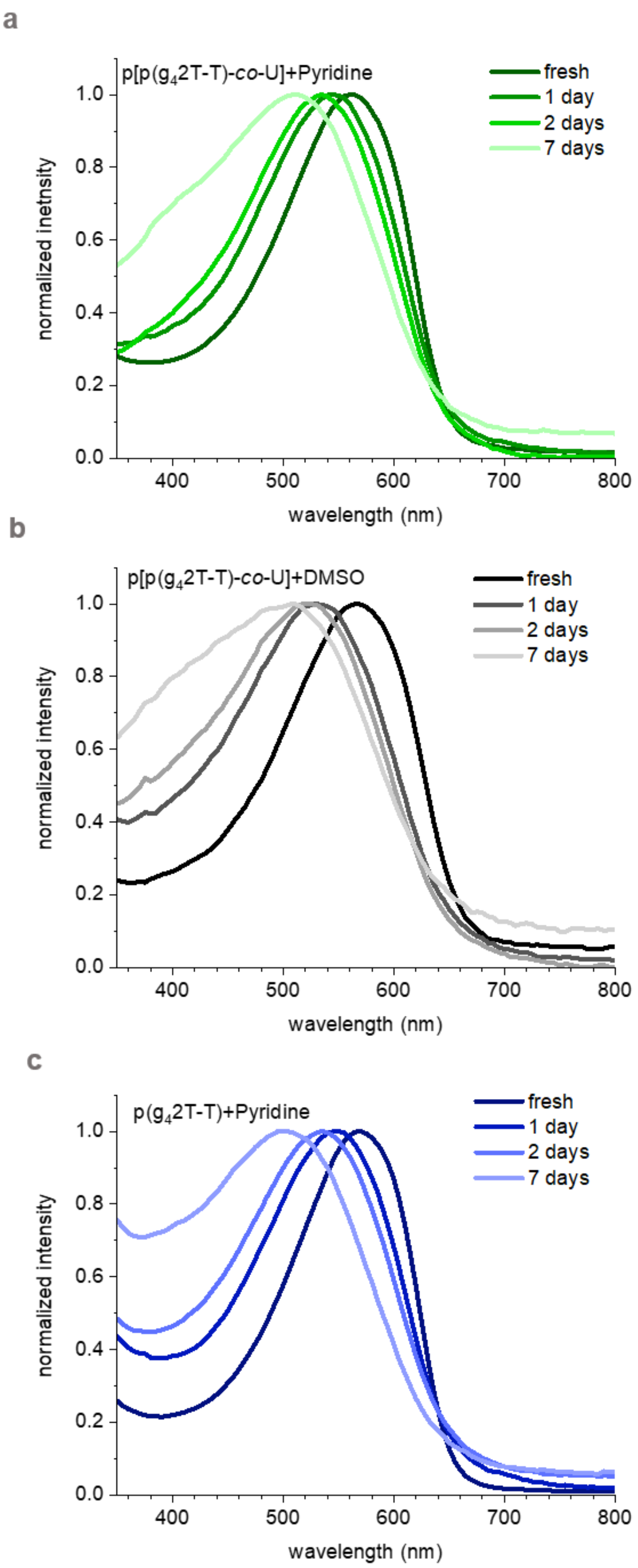

Figure S3. Normalized UV-vis absorbance spectra of p[p($g_4$2T-T)-*co*-U] in (a) pyridine, (b) DMSO, and (c) p($g_4$2T-T) in pyridine. Samples were stored in closed vials at room temperature.

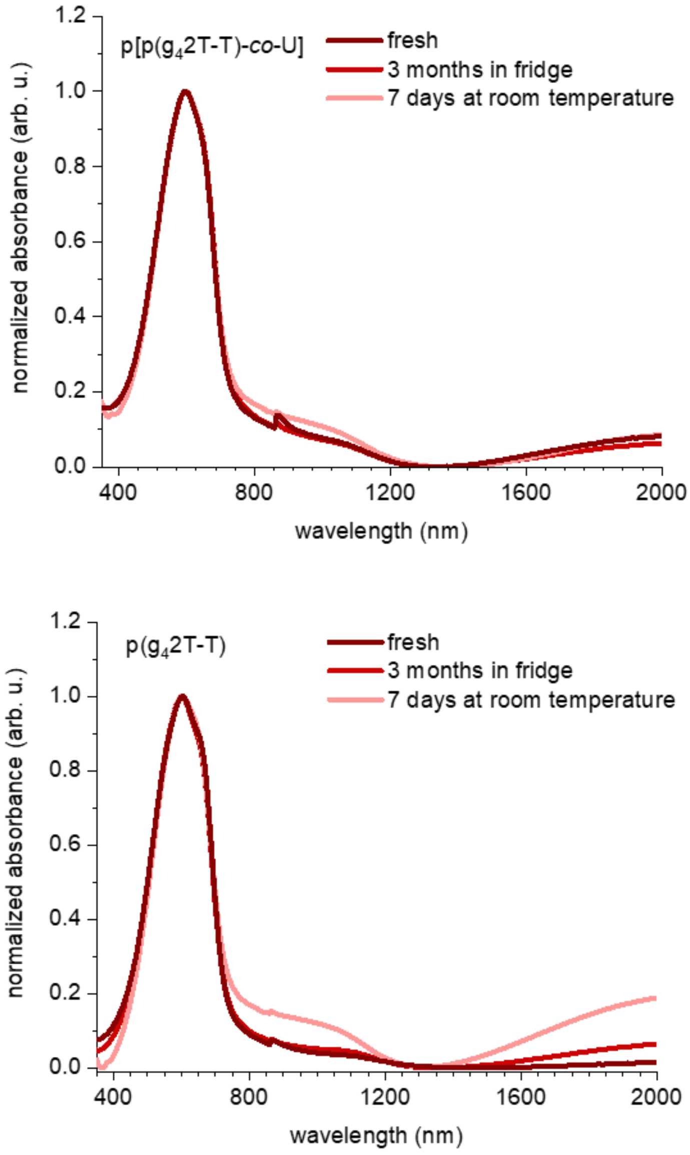

Figure S4. Normalized UV-vis absorbance spectra of (top) p[p($g_4$2T-T)-*co*-U] and (bottom) p($g_4$2T-T), spin-coated from pyridine. Films were stored in a fridge at 5 °C over 3 months and subsequently kept at room temperature for 1 week. The polymer absorption peak remains unchanged, while polaronic absorption peaks in the NIR appear.

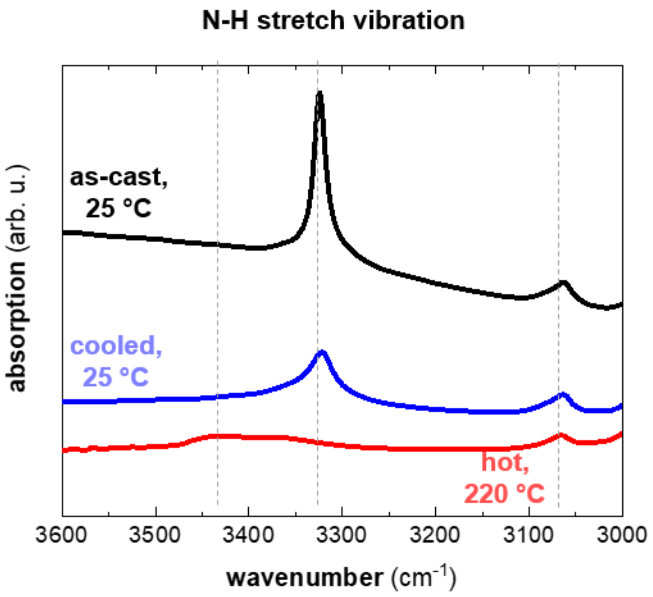

Figure S5. FTIR of the urethane N-H stretch vibration recorded at 25 °C for a p[p($g_4$2T-T)-*co*-U] film cast from DMSO (black), recorded for the same film at 220 °C (red) and after cooling from 220 to 25 °C (blue). The absence of an absorption peak at 3325 cm^{-1} at 220 °C and emergence of a peak at higher wavenumber is consistent with the dissociation of hydrogen bonds upon heating.

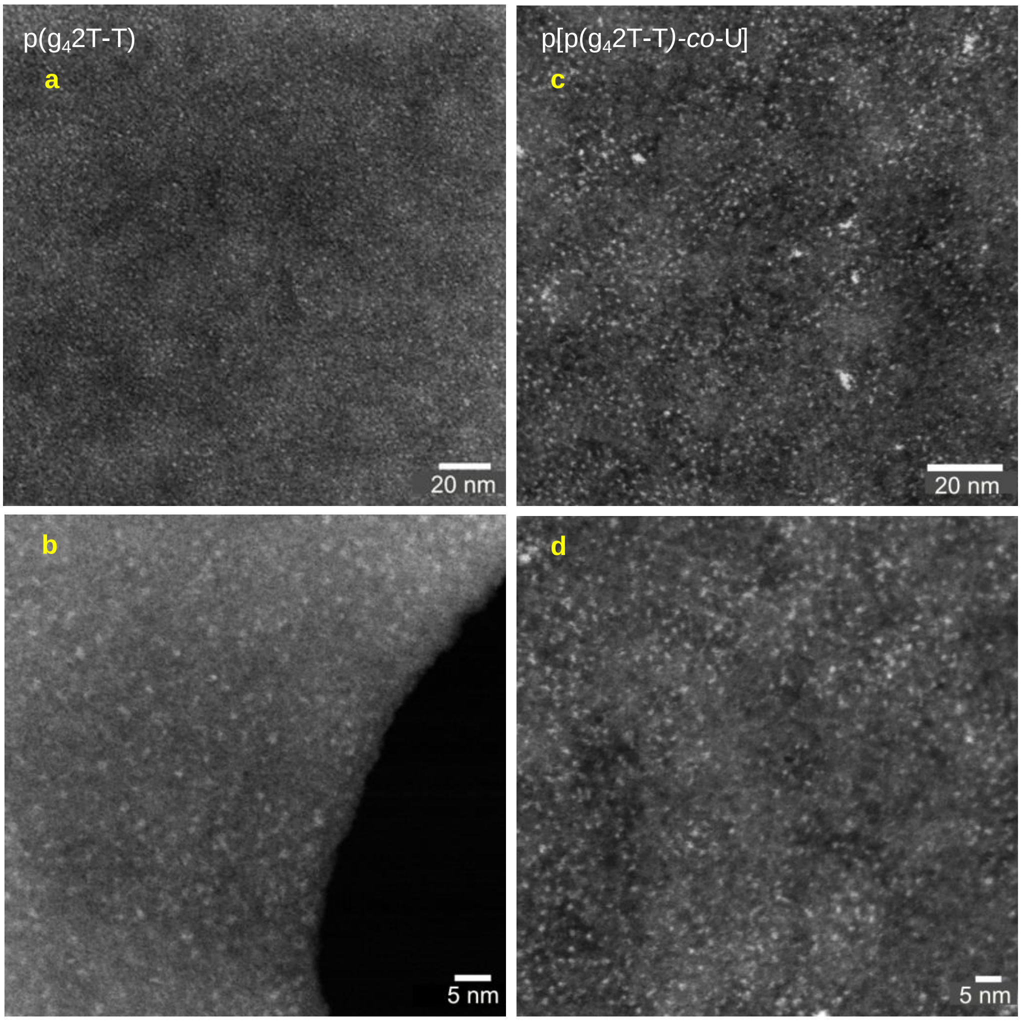

Figure S6. High-angle annular dark-field scanning transmission electron microscopy (HAADF-STEM) images of (a, b) p($g_4$2T-T) and (c, d) p[p($g_4$2T-T)-*co*-U] (n=1). Both materials display the same granular texture.

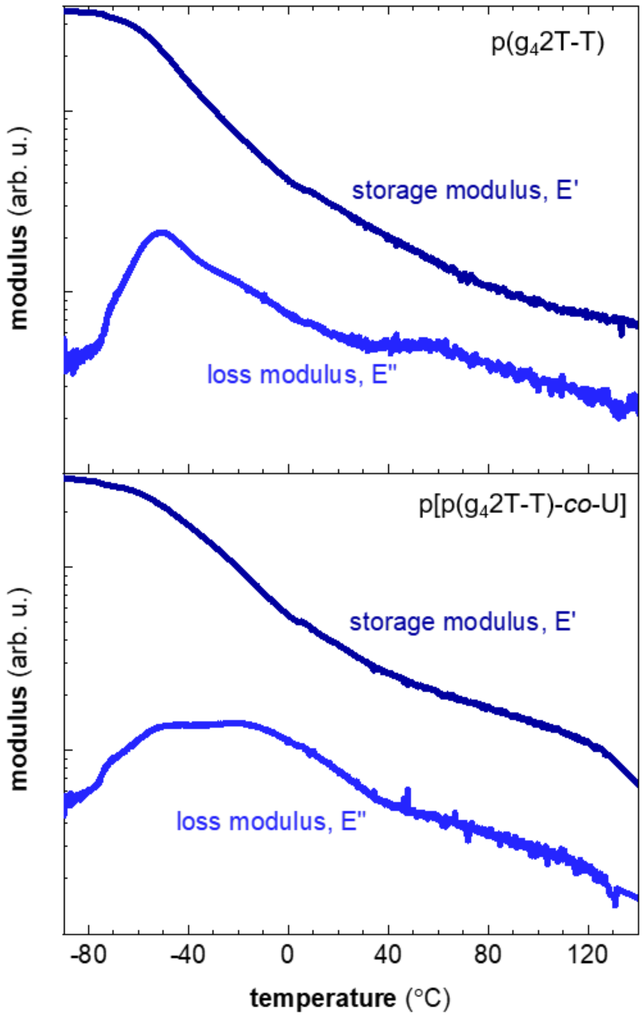

Figure S7. Storage and loss modulus of (a) p($g_4$2T-T) and (b) p[p($g_4$2T-T)-*co*-U] reinforced with a glass mesh as a function of temperature, measured with DMA at 1 Hz. The T_g transitions are clearly observed at -50 °C for p($g_4$2T-T) and at -44 °C and -20 °C for p[p($g_4$2T-T)-*co*-U].

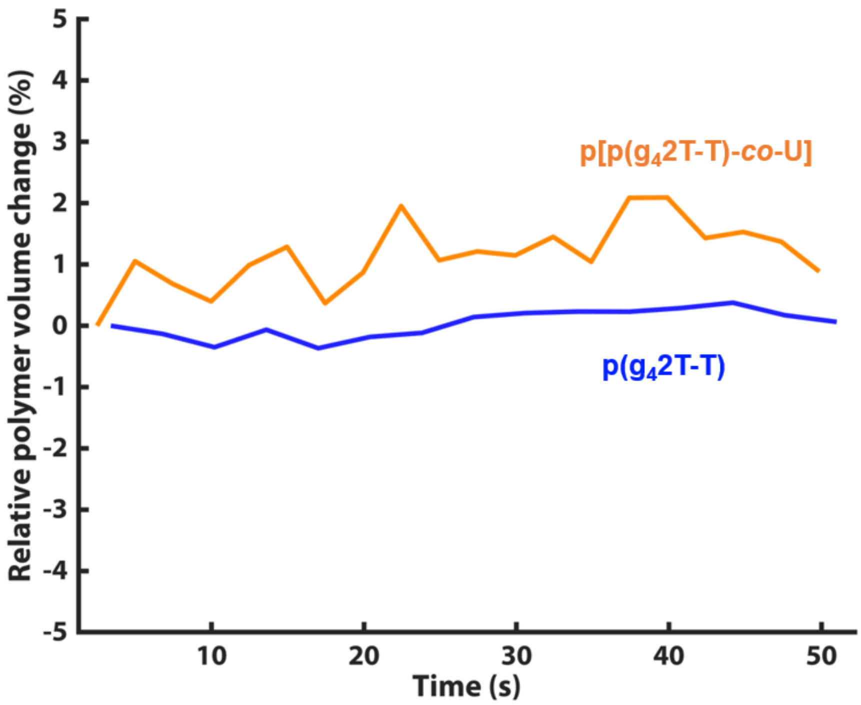

Figure S8. Passive swelling of the polymer coatings when immersed in 0.01M KCl electrolyte.

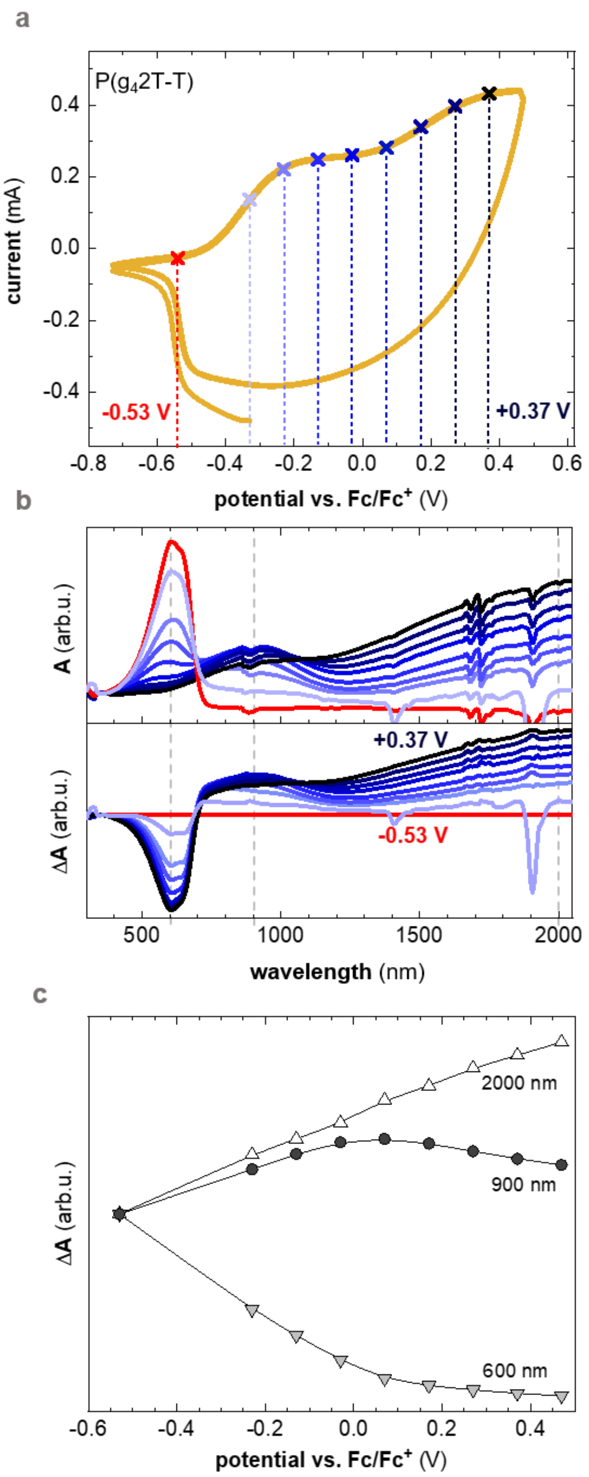

Figure S9. (a) Cyclic voltammogram of p($g_4$2T-T) in 0.1 M solution of [EMIM][BF_4]; (b) UV-vis-NIR absorbance spectra recorded at each applied electrochemical potential (top) and difference in absorbance ΔA between neutral and oxidized thin films (bottom); (c) ΔA at 600 nm, 900 nm and 2000 nm vs. oxidation potential.

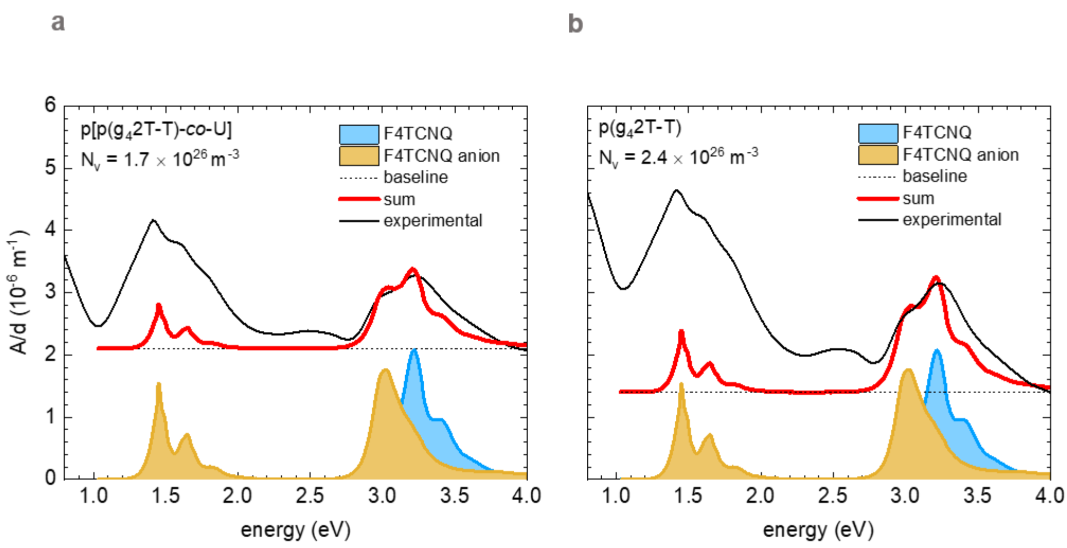

Figure S10. Film thickness normalized UV-vis-NIR absorbance spectra of (left) p[p($g_4$2T-T)-*co*-U] and (right) p($g_4$2T-T) sequentially doped with F4TCNQ (black), spectra of neat F4TCNQ (blue) and the F4TCNQ anion (yellow) from ref. [1], and best fits in the UV region (red) including a horizontal baseline (dashed black).

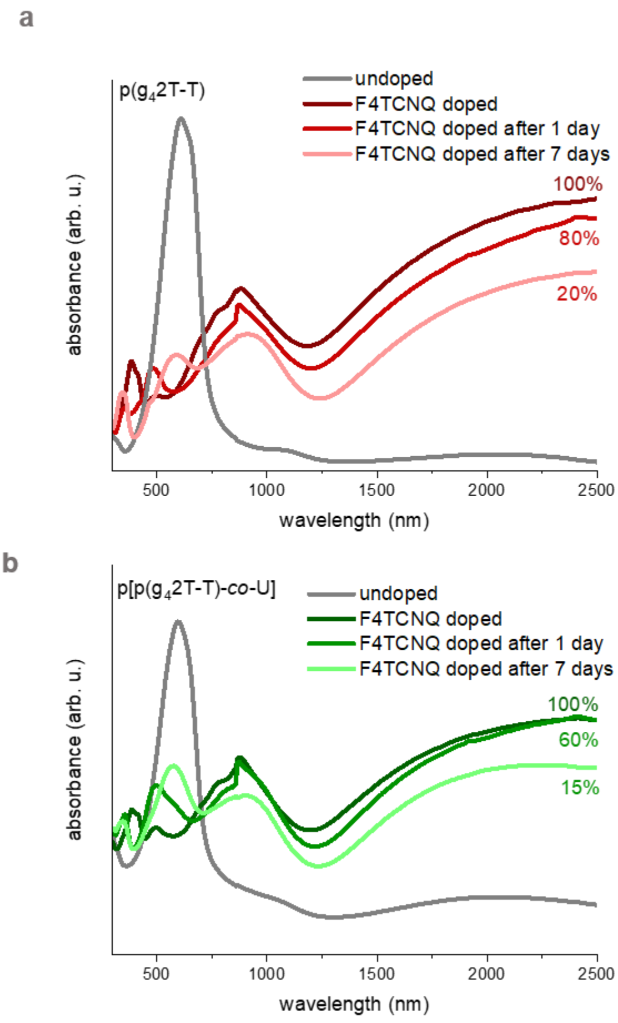

Figure S11. UV-vis-NIR absorbance spectra recorded for (a) p($g_4$2T-T) and (b) p[p($g_4$2T-T)-*co*-U] thin films before and immediately after doping with F4TCNQ, as well as of the doped films after 1 and 7 days at room temperature in the dark. The polaronic absorption peaks decrease and the polymer absorption peak, which diminishes upon doping, starts to rise again with time. The numbers next to each spectrum correspond to the ratio $\sigma/\sigma_0 \cdot 100\%$, where σ is the electrical conductivity of the sample and σ_0 the conductivity measured directly after doping (σ_0 = (27 ± 1) S cm^{-1} for homopolymer; σ_0 = (17 ± 6) S cm^{-1} for copolymer). Films were spin-coated from 80-90 °C hot solutions of the polymers in anhydrous pyridine (10 g L^{-1}) onto hot glass slides (homopolymer film thickness = 30 nm; copolymer film thickness = 50 nm). Sequential doping was done at room temperature by drop-casting a solution of F4TCNQ in AcN (10 g L^{-1}) onto the polymer films, followed by spinning off the solution after 1 min.

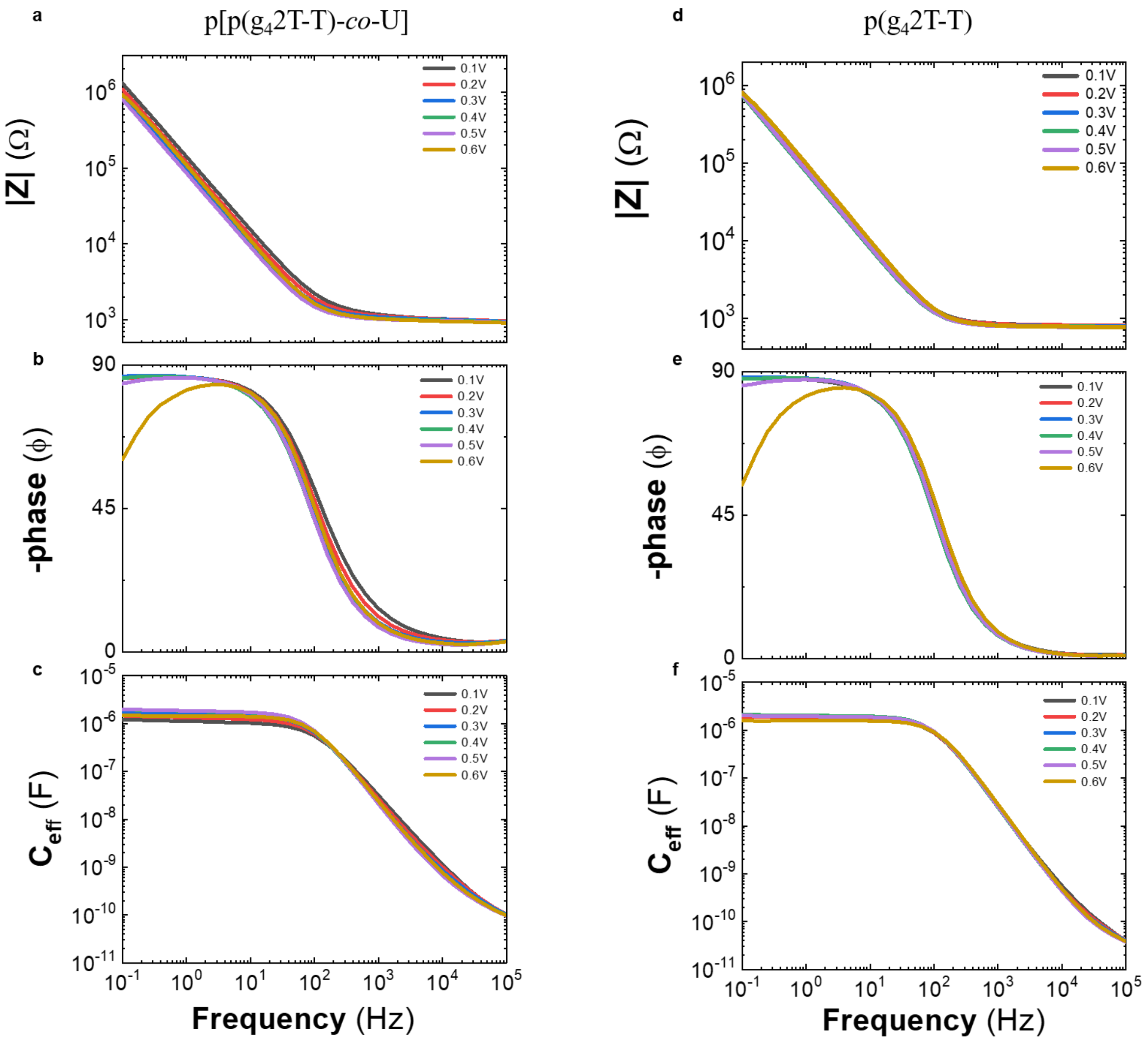

Figure S12. Electrochemical Impedance Spectroscopy: (a) impedance, (b) phase angle, and (c) effective capacitance for p[p(g42T-T)-*co*-U], and (d-f) likewise for p(g42T-T); all collected with a 10 mV sinusoidal small signal superimposed on offsets from 0.1 to 0.6 V (offset = -V_g).

Synthetic procedures

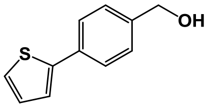

(4-(thiophen-2-yl)phenyl)methanol (1). $Pd_2(dba)_3$ (6 mg, 6.6 μmol), (*o*-tol)$_3$P (9 mg, 29.6 μmol) and toluene (5 mL, anhydrous & degassed) were added to a dry flask under nitrogen atmosphere and stirred at 80 °C for 5 minutes. Then, 2-(tributylstannyl)thiophene (2.2g, 5.89 mmol) and 4-bromobenzyl alcohol (1.24 g, 6.6 mmol) were successively added after which the reaction was continued for 16 hours. The resulting reaction mixture was evaporated to dryness and passed through a short silica plug with ethyl acetate as the eluent. Recrystallization of the compound from heptane afforded the title compound (0.85 g (1st crop) and 0.1 g (2nd crop), total yield 82%) as off-white crystals. ^{1}H NMR (400 MHz, Chloroform-*d*) δ 7.65 – 7.57 (m, 2H), 7.44 – 7.34 (m, 2H), 7.31 (dd, *J* = 3.6, 1.2 Hz, 1H), 7.28 (dd, *J* = 5.1, 1.2 Hz, 1H), 7.08 (dd, *J* = 5.1, 3.6 Hz, 1H), 4.71 (d, *J* = 5.9 Hz, 2H), 1.65 (t, *J* = 6.0 Hz, 1H). ^{13}C NMR (101 MHz, Chloroform-*d*) δ 144.06, 140.07, 133.77, 128.03, 127.54, 126.06, 124.82, 123.12, 64.96.

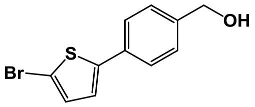

(4-(5-bromothiophen-2-yl)phenyl)methanol (2). Compound 1 (200 mg, 1.05 mmol) was dissolved in a mixture of chloroform:acetic acid (3 mL, 1:1 v/v) and stirred at 0 °C for 15 min under nitrogen atmosphere. Then, NBS (187 mg, 1.05 mmol) was added in small portions after which the reaction was continued for 2 hours at 0 °C and 1 hour at room temperature. Chloroform was added to the reaction after which the organic phase was successively washed with a saturated solution of $Na_2S_2O_3$, a saturated solution of $NaHCO_3$ and brine. The organic phase was dried over $MgSO_4$, filtered and the solvent was removed *in vacuo*.

Recrystallization of the obtained crude material from heptane afforded the title compound (190 mg (1st crop) and 70 mg (2nd crop), total yield 89%) as off-white crystals. ^{1}H NMR (400 MHz, Chloroform-*d*) δ 7.54 – 7.48 (m, 2H), 7.41 – 7.33 (m, 2H), 7.05 (d, *J* = 3.9 Hz, 1H), 7.03 (d, *J* = 3.9 Hz, 1H), 4.71 (d, *J* = 5.9 Hz, 2H), 1.66 (t, *J* = 5.9 Hz, 1H). ^{13}C NMR (101 MHz, Chloroform-*d*) δ 145.49, 140.53, 133.01, 130.84, 127.59, 125.76, 123.25, 111.39, 64.93.

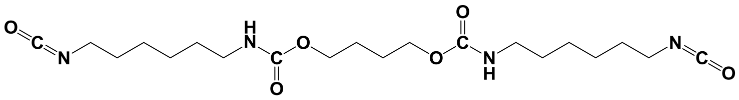

butane-1,4-diyl bis((6-isocyanatohexyl)carbamate) (3). To a flame-dried and pre-weighed 1-necked round-bottom flask equipped with a septum, 1,4-butanediol (100 mg, 1.11 mmol), 1,6-hexyldiisocyanate (2.2 g, 13.3 mmol) and one drop of dibutyltindilaurate were added and reacted for two hours at room temperature under nitrogen atmosphere. A white precipitate was formed as the reaction progressed. After two hours, anhydrous heptane was added to the stirred reaction mixture to remove excess 1,6-hexyldiisocyanate, the white precipitate allowed to settle and the supernatant removed with a syringe. The heptane wash was repeated two more times after which the reaction product was dried *in vacuo* and obtained as a white, waxy solid (425 mg, 90 %) of which part was directly used for the next step. ^{1}H NMR (400 MHz, DMSO-d_6) δ 7.05 (s, 2H), 3.93 (s, 4H), 3.50 – 3.20 (m, 4H), 2.94 (t, *J* = 6.4 Hz, 4H), 1.68 – 1.47 (m, 8H), 1.46 – 1.16 (m, 12H).

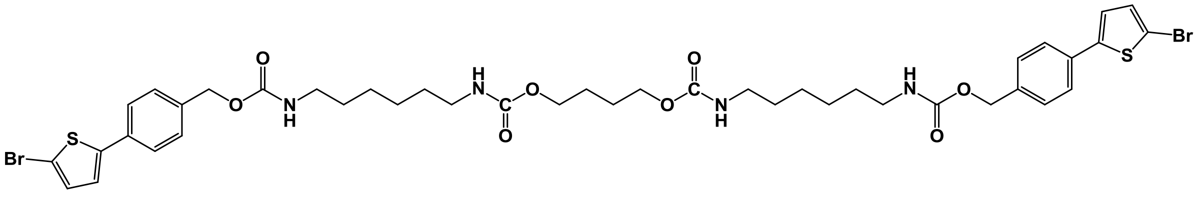

4-(5-bromothiophen-2-yl)benzyl (1-(4-(5-bromothiophen-2-yl)phenyl)-3,12-dioxo-2,13-dioxa-4,11-diazaheptadecan-17-yl) hexane-1,6-diyldicarbamate (4). To a flame-dried 1-necked round-bottom flask compound **3** (127.95 mg, 0.3 mmol) and compound **2** (167.2 mg, 0.62 mmol) were added after which the flask was subjected to three nitrogen/vacuum flush cycles. Then, anhydrous DMF was added, the reaction mixtures heated to 50 °C to form a clear solution and subsequently reacted for 1 hour. A white precipitate was formed during the reaction. The reaction mixture was cooled to room temperature, diluted with diethyl ether and the product was filtered off. The obtained white powder was subsequently stirred in anhydrous DMF at 80 °C to remove traces of compound 3, cooled to room temperature and washed with diethyl ether to remove residual DMF. Then, the product was dissolved in 16 mL of anhydrous DMSO at ~120 °C, slowly cooled (a precipitate forms at ~80 °C) to room temperature again after which the white precipitate was filtered off, rinsed with acetone to remove traces of DMSO and dried *in vacuo* to afford the title compound as a white powder (0.22 g, 76%). ^{1}H NMR (400 MHz, DMSO-d_6) δ 7.58 (d, J = 8.0 Hz, 4H), 7.44 – 7.30 (m, 4H + 2H), 7.29 – 7.15 (m, 2H + 1H), 7.02 (s, 2H), 4.98 (s, 4H), 3.96 – 3.80 (m, 4H), 3.05 – 2.81 (m, 8H), 1.64 – 1.50 (m, 4H), 1.48 – 1.29 (m, 8H), 1.29 – 1.11 (m, 8H). HRMS (ESI) theoretical m/z: 985,1486 (M+Na); found: 985,1463 (M+Na).

p[p(g$_4$2T-T)-*co*-U]. To a dry two-necked roundbottom flask, 13,13'-((5,5'-dibromo-[2,2'-bithiophene]-3,3'-diyl)bis(oxy))bis(2,5,8,11-tetraoxatridecane) (250 mg, 0.334 mmol),[1] 2,5-bis(trimethylstannyl)thiophene (156.02 mg, 0.38 mmol), $Pd_2(dba)_3$ (6 mg, 2 mol%) and (*o*-tol)$_3$P (8 mg, 4 mol%) were dissolved in THF (8 mL, dry, degassed by N_2 purging for 30 min) and reacted for 2 hours at 65 °C during which the reaction turned from slightly yellow to deep red. Then, **compound 4** (45.1 mg, 0.0467 mmol) and DMSO (anhydrous, degassed by N_2 purging for 30 min) were added to the reaction mixture. A solvent exchange was

performed by increasing the reaction temperature to 80 °C while purging with N_2, after which the reaction was continued for 48 hours. The resulting deep purple, viscous solution was precipitated in isopropanol, filtered and dried for 48 hours *in vacuo*. Then, the polymer was redissolved in dry DMSO at 60 °C and vigorously stirred with 100 mg sodium diethyl dithiocarbamate for one hour. The obtained mixture was precipitated in isopropanol, filtered into a thimble and extracted with isopropanol, diethyl ether and dichloromethane until the washings were clear. The polymer was collected by cutting the thimble into pieces which were stirred in DMSO at 80 °C. The obtained mixture was filtered through a packed cotton wool plug into diethyl ether, resulting in a blue precipitate. After filtration, extensive washing of the polymer with diethyl ether and drying *in vacuo*, the polymer was obtained as a blue powder (120 mg, 46%).

Estimate of the T_g of p(g$_4$2T-T)

We attempted to predict the T_g of p(g$_4$2T-T) by following the rationale recently proposed by Xie et al.,[2] which estimates the mobility of a repeat unit by summing the mobility of each atom normalized by the total number of atoms. The mobility of the repeat unit is given by:

$$\zeta = \frac{\sum \zeta_i N_i}{\sum N_i} = \frac{\zeta_{thiophene} N_{thiophene} + \zeta_{flexible} N_{flexible}}{N_{thiophene} + N_{flexible}} \quad (1)$$

where N_i is the number of atoms (excluding hydrogens) that belong to a thiophene ring, $N_{thiophene}$, or an atom in a flexible side chain, $N_{flexible}$. ζ_i is a measure of the atomic mobility of each corresponding unit. Xie et al. have set $\zeta_{flexible} = \zeta_{C-C} = \zeta_{C-O} = 1$ and $\zeta_{thiophene} = 0.72$, which yields for p(g$_4$2T-T) a value of $\zeta = 0.902$. The T_g is then given by the empirical relationship:

$$T_g/°C = 979 - 1102\zeta \quad (2)$$

For p(g$_4$2T-T) we obtain T_g = -15 °C.

1. R. Kroon, D. Kiefer, D. Stegerer, L. Yu, M. Sommer, C. Müller, *Adv. Mater.* **2017**, *29*, 1700930.

2. R. Xie, A. R. Weisen, Y. Lee, M. A. Aplan, A. M. Fenton, A. E. Masucci, F. Kempe, M. Sommer, C. W. Pester, R. H. Colby, E. D. Gomez, *Nat. Commun.* **2020**, *11*, 893.
